# Thyrolipoma: A Rare Presentation of the Thyroid Gland

**DOI:** 10.7759/cureus.45310

**Published:** 2023-09-15

**Authors:** Ankita Gyanchandani, Samarth Shukla, Sunita Vagha, Sourya Acharaya, Rinkle Gemnani

**Affiliations:** 1 Department of Pathology, Jawaharlal Nehru Medical College, Datta Meghe Institute of Higher Education and Research, Wardha, IND; 2 Department of Medicine, Jawaharlal Nehru Medical College, Datta Meghe Institute of Higher Education and Research, Wardha, IND

**Keywords:** adipose tissue, thyroidectomy, goitre, thyrolipoma, lipoadenoma, neck swelling

## Abstract

Thyrolipoma is a rare disorder marked by substantial adipose tissue growth inside the thyroid gland. Fatty tissue is frequently seen in salivary glands, thymus, breasts, and pancreas but rarely in the thyroid gland. The fascinating and unusual illness known as thyrolipoma presents challenging diagnostic and therapeutic issues. Due to the rarity of thyrolipoma, doctors must evaluate thyroid nodules with a high index of suspicion, especially those who are radiologically and clinically worrisome. We present a study of a 50-year-old female who came with the complaint of midline neck mass for one year. On clinical examination, a diagnosis of multinodular goiter was made. Computed tomography (CT) scan was suggestive of a heterogenous enhanced thyroid mass lesion. Thus, a total thyroidectomy was performed. On histopathological examination, a final diagnosis of thyrolipoma was made. The abstract aims to provide an overview of the clinical presentation, diagnosis, and treatment options of thyrolipoma, as well as highlight the importance of early recognition and appropriate management of this rare tumor.

## Introduction

Thyroid tissue and fat can combine to form a benign tumor called thyrolipoma, often referred to as a lipoadenoma or an adenolipoma. Its genesis is uncertain, but few writers assert that it is an abnormality that arises either during thyroid encapsulation or as a result of hypoxia-induced fibroblast metaplasia. Adipose tissue is frequently seen in salivary glands, thymus, breasts, and pancreas but is uncommon to locate it in the thyroid gland. Only a few numbers of thyroid nodules with fat inclusions have been reported in the literature [1].

Depending on whether they include fat, neoplastic, and non-neoplastic thyroid lesions can be categorized into two categories. Thyrolipoma of the thyroid gland is a commonly occurring fat-containing lesion [2,3]. It is differentiated by the presence of mature fatty tissue dispersed throughout the tumor; also, it is assumed to be a variant of follicular adenoma. Additionally, a few cases of adipose cell involvement in papillary carcinoma and follicular malignancy have been recorded [4]. Even while these tumors are typically tiny and asymptomatic, they can occasionally enlarge significantly, compress adjacent structures, and produce symptoms including trouble breathing or eating [5]. Although uncommon, thyrolipoma must be recognized as early as possible to give the right management and guarantee the best possible patient results.

## Case presentation

**Figure 1:**
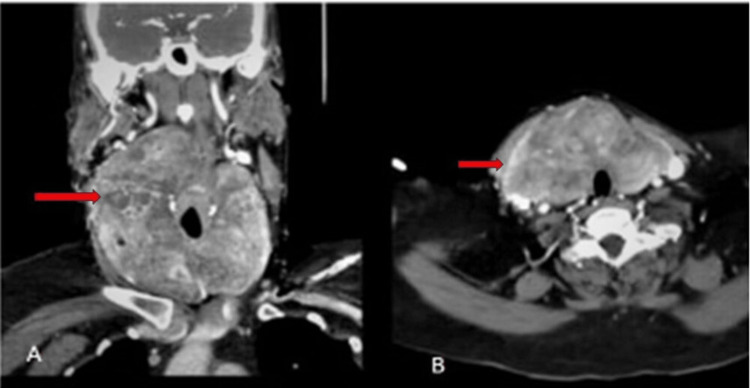
CT showing enhanced thyroid mass lesion. (A) Coronal CT showing heterogeneous enhanced thyroid mass lesion (red arrow) along with compression of the pharynx. (B) Axial CT also showing enhanced thyroid mass lesion (red arrow) along with compression of the pharynx, and the trachea is slightly deviated to the left side.

A 50-year-old female was seen with a single, growing midline neck swelling during the previous 12 months. There was no history of palpitations, tremors, sweating, shortness of breath, irregularities in the menstrual cycle, difficulty in swallowing, or hoarseness. Clinical examination showed a significant midline neck edema. The swelling moved on swallowing. Thyrotropin, triiodothyronine, and thyroxine levels were found to be normal. Diagnosis of diffuse multinodular goiter was made during a clinical examination. A computed tomography (CT) scan showed a heterogeneous enhanced thyroid mass lesion as shown in Figures 1A, 1B.

**Figure 2:**
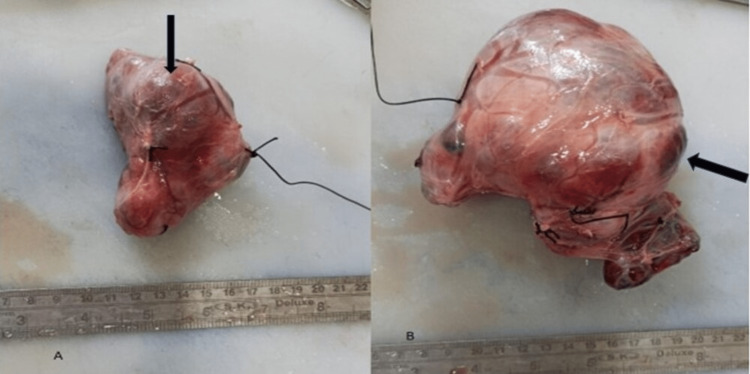
Right and left lobes of the thyroid. (A) Right lobe of the thyroid and (B) left lobe of the thyroid showing diffuse brownish surface with nodular exterior appearance (black arrow).

There were no visible swollen lymph nodes. The complete thyroid gland was removed. The left lobe measured 12 x 7 cm, while the right lobe was 10 x 9 cm in length. The texture of both lobes was delicate. The surface was diffuse brownish with a nodular exterior appearance as shown in Figures 2A, 2B.

**Figure 3:**
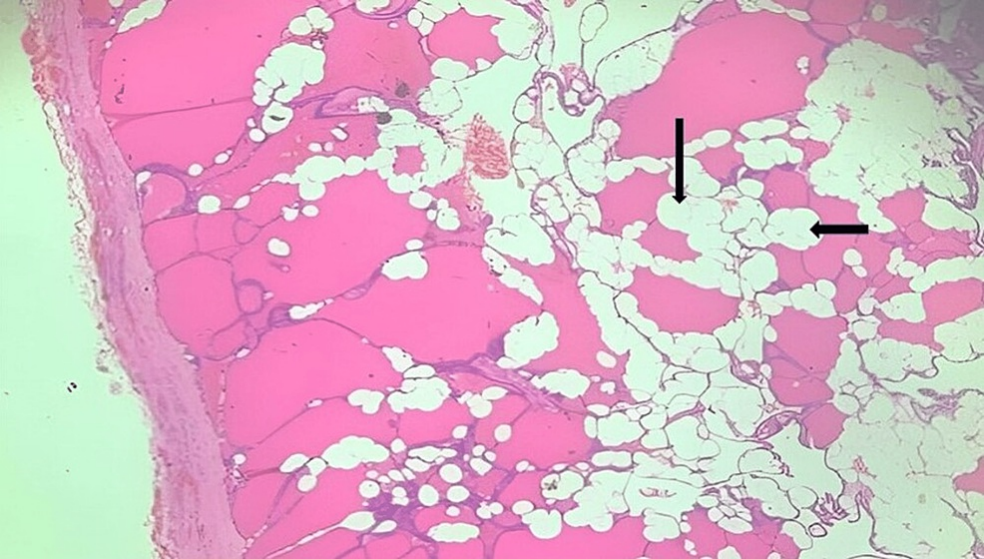
Histopathological slide (H and E (hematoxylin and eosin) stain, 10x) showing a small group of adipocytes laying between colloid-filled thyroid follicles (black arrows), features suggestive of thyrolipoma.

On microscopic examination, lobules of follicles filled with colloid, lined by cuboidal cells separated by fibrous strands. Along with this, mature adipose tissue was present in the interfollicular stroma with no cytological atypia. The vascular or capsular invasion was not evident. Histopathological features were suggestive of thyrolipoma as shown in Figure 3.

## Discussion

Clinically, thyrolipoma is identical to its typical counterparts. They mostly result in neck volume growth and compressive sensations. Typically, the patients are euthyroid. In this study, the case had normal T3, T4, and thyrotropin along with neck swelling. Clinically, a diagnosis of multinodular goiter was made. In our case, on radiological examination, the mass was compressing adjacent structures and slightly deviating the trachea, and thus, total thyroidectomy was performed. Thyroid glands with adjacent capsules, surrounding arteries, and connective tissue rarely have small quantities of adipose tissue. The adipose tissue has been noted in neoplastic situations like adenoma, papillary carcinoma, and sporadically liposarcoma, as well as non-malignant conditions like nodular hyperplasia, amyloid goiter, Graves’ disease, lymphocytic thyroiditis, and thyroid atrophy. Thyrolipoma is a well-defined, encapsulated mass lesion that differs from widespread thyroid lipomatosis and amyloid goiter with fat cells [5].

Uncertainty surrounds the thyroid glands’ adipose tissue's genesis. According to some experts, the presence of intrathyroidal fat is a developmental abnormality caused by adipose tissue trapping during the encapsulation of the thyroid. Others claim that fibroblasts with prolonged hypoxia have a metaplastic origin. Gill et al. described a case of thyrolipoma that was concurrent with thymolipoma and pharyngeal lipoma and remarked that this suggested a problem with foregut development [6].

The simultaneous occurrence of these tumors revealed a problem with the primitive foregut's development, according to Breek et al. [4], who described a study of thyrolipoma and thymolipoma. They also highlighted that the thyroid and thymus arise from the primitive foregut [4]. The metaplastic theory has both its supporters and refuters. Schröder et al. [5] think that stromal fibroblasts are the source of fat cells because persistent tissue hypoxia is thought to be the cause of the existence of fatty tissue in amyloid goiters. However, it is asserted that massive steatosis of follicular cells, as seen in the lipid-rich type of clear-cell thyroid adenomas and carcinomas, is most likely the product of neoplastic thyrocytes undergoing metaplastic transformation [5].

In addition to heterotopic nests of adipocytes, diffuse lipomatosis, adenolipoma, amyloid goiter, lymphocytic thyroiditis, intrathyroidal or parathyroid lipoma, encapsulated papillary cancer, and liposarcoma are among the possible diagnoses for the presence of mature adipose tissue in the thyroid. While thyroid lipomatosis is a diffuse condition, thyrolipoma is a well-defined, encapsulated nodule made up of thyroid follicles combined with mature adipose tissue [7,8] similar to how its diffuse nature sets it apart from heterotrophic adipocyte nests that are primarily seen in subcapsular regions. Using a particular dye and polarizing microscopy, one may distinguish an amyloid goiter from one that may also contain fat cells [9,10]. Lymphocytic thyroiditis might be accompanied by fat infiltration. When lobulated adipose tissue and follicular forms of parathyroid chief cells are found in the stroma, the differential diagnosis of parathyroid lipoma should also be taken into account. Our case demonstrates the significance of evaluating patients with thyroid adiposity to rule out other diseases, particularly malignant ones, that manifest as diffuse gland enlargement. Kalluri et al. reported a case of thyroid lipomatosis in a patient of post-renal transplantation with no evidence of amyloid deposition or papillary carcinoma [7].

## Conclusions

In conclusion, thyrolipoma is a rare benign tumor that arises from the thyroid gland. It is composed of both thyroid follicular cells and adipose tissue. While it is often asymptomatic, it causes compression symptoms if it is large enough. The diagnosis is made by a combination of clinical, radiological, and histopathological evaluation. Treatment is usually surgical removal of the tumor, and the prognosis is excellent with no reported cases of malignant transformation. However, long-term follow-up is needed to monitor for the recurrence or development of new lesions. Overall, while rare thyrolipoma is an important differential diagnosis to consider in cases of thyroid nodules, the need for surgery in these situations must be re-evaluated through additional research using cytological criteria.
